# Spotted Patterns of Glass Frogs Can Disguise Imperfections in Transparency to Improve Camouflage

**DOI:** 10.1002/ece3.74623

**Published:** 2026-11-02

**Authors:** Justin Yeager, Leonardo Ávila, James B. Barnett

**Affiliations:** ^1^ Grupo de Investigación en Biodiversidad, Medio Ambiente y Salud (BIOMAS), Facultad de Ingenierías y Ciencias Aplicadas Universidad de Las Américas Quito Ecuador; ^2^ Dirección de Investigación y Vinculación, Escuela de Biotecnología, Facultad de Ingenierías y Ciencias Aplicada Universidad de Las Américas Quito Ecuador; ^3^ School of Natural Sciences Trinity College Dublin Dublin Ireland

**Keywords:** camouflage, Centrolenidae, crypsis, glass frogs, transparent camouflage

## Abstract

Transparency could be considered the idealized version of camouflage, capable of producing effective concealment across heterogeneous habitats. However, although examples of highly transparent species do exist, ‘transparent’ species are highly variable and are often either only partially transparent or better described as translucent. Exactly how these different forms of ‘imperfect’ transparency may act as camouflage remains largely unknown. The glass frogs (Centrolenidae) have long been thought of as a classic example of transparent camouflage due to their see‐through ventral skin. Yet even in these seemingly exemplary cases, glass frogs are better described as translucent due to the presence of obligate opaque structures, such as the internal organs, within the body and opaque color patterns in their dorsal skin. These opaque patterns could be predicted to detract from transparency, with the potential to increase detectability and therefore predation risk. One potential explanation is that the addition of opaque patterning could help to conceal the internal organs from view while maintaining the cryptic effects of translucency. Here we tested whether the presence of opaque spots could reduce predation risk for translucent frogs by presenting model frogs, which differed in opacity and dorsal patterning, to wild birds and recording the rate at which these models were attacked. We found that the addition of opaque dorsal patterning reduced predation risk but there was no effect of translucency on survival. These data suggest that opaque patterning in glass frogs may help to conceal conspicuous internal structures but that partial translucency does not necessarily guarantee effective concealment. Future work is needed to explore the full range of dorsal patterning found in translucent glass frogs and to further examine how patterns comprised of both opaque and transparent elements may function as camouflage in a wider range of taxa.

## Graphical abstract

Transparency could be considered the idealized version of camouflage, capable of producing remarkable background matching across heterogeneous microhabitats. However, although examples of highly transparent species indeed exist, transparency is often imperfect in execution, especially in terrestrial animal species. Our study suggests that opaque color patterning in glass frogs, which seemingly reduces transparency, actually helps to conceal conspicuous internal structures, improving camouflage via blending elements of opaque and transparent camouflage.

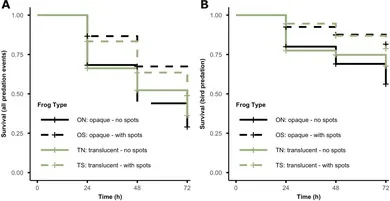

## 1 Introduction

Camouflage is a common set of anti‐predatory strategies whereby species prevent detection and/or recognition by blending in with their surroundings (Merilaita et al. 2017; Cuthill 2019). This concealment can be achieved using opaque pigments or structural colors which can, for example, replicate the background or break up the organism's outline (Merilaita et al. 2017; Cuthill 2019), or via transparency where the background remains visible to varying degrees through the body (Johnsen 2001; Bagge 2019; Barnett et al. 2025). As the substrate itself is visible, transparency can, in theory, result in particularly high‐precision background matching (Johnsen 2001; Bagge 2019; Barnett et al. 2025). However, the degree of concealment may be limited by the ability to produce effective transparency across significant portions of the body (Johnsen 2001; Bagge 2019; Barnett et al. 2025).

The glass frogs (Centrolenidae) represent a remarkable example of transparent camouflage with their (often) transparent ventral skin permitting a view of the internal organs (Guayasamin et al. 2009, 2020; Barnett et al. 2020; Taboada et al. 2022). However, although glass frogs are frequently hailed as being highly transparent, levels of transparency vary greatly, ranging from what may be considered some of the most transparent terrestrial vertebrates (e.g., *Hyalinobatrachium* spp.) to species which are nearly completely opaque (e.g., *Sachatamia orejuela* ) (Guayasamin et al. 2009, 2020). This variance has triggered recent interest in understanding the evolution of transparency and the ecological and physiological processes underpinning diversity in both form and function (Guayasamin et al. 2020; Barnett et al. 2020, 2025; Taboada et al. 2022).

At first glance, glass frogs may be expected to be under strong selection to become increasingly transparent as any defects in transparency could increase their detectability against the background by diminishing the efficacy of camouflage. Indeed, *Hyalinobatrachium* spp. have specialist adaptations which increase transparency, including storing their red blood cells in the liver when at rest (Taboada et al. 2022). However, our understanding of how and why transparency has evolved is still limited (Johnsen 2001; Bagge 2019; Barnett et al. 2025) and more work is needed to explain the diversity found in both transparency and in opaque patterning found across glass frog species (Guayasamin et al. 2009, 2020).

Indeed, despite many species of glass frog having transparent ventral skin, the dorsal skin is pigmented such that these frogs are best described as a translucent green (Guayasamin et al. 2009, 2020; Barnett et al. 2020; Taboada et al. 2022). This ‘imperfect transparency’ still has advantages as, although color does not change between backgrounds, the brightness of the dorsum does appear to change according to the substrate (Barnett et al. 2020; Guayasamin et al. 2020). As these frogs are predominantly found on living green leaves, this green‐biased translucency may be sufficient to effectively conceal glass frogs against a wide range of natural substrates (Barnett et al. 2020; Guayasamin et al. 2020; Angiolani‐Larrea et al. 2025). Moreover, by surrounding the more opaque center of the body with the more translucent legs, glass frogs may disguise their recognizable outline and reduce edge contrast against the substrate (i.e., edge diffusion; Webster et al. 2015; Barnett et al. 2020). However, the presence of obligate opaque structures within the body (i.e., the internal organs and bones) raises the question of how physiological constraints may affect the evolution of transparency and whether additional dorsal pigmentation may also play an important role in concealment (Yeager et al. 2026).

In a previous study, we conducted a field predation experiment with model frogs and wild birds to examine how the presence and shape of opaque internal structures may affect glass frog survival (Yeager et al. 2026). These model frogs included patterns of green‐tinted white clay which were reminiscent of the viscera, which in glass frogs is frequently contained within a reflective, white sac (Cisneros‐Heredia and Mcdiarmid 2007; Taboada et al. 2022). We predicted that surrounding the opaque regions with areas of translucency would reduce detectability and predation risk. However, we found no significant effect of opacity on the rate of predation (Yeager et al. 2026), suggesting that the presence of internal structures had negated the camouflaging effect of transparency (Barnett et al. 2020).

The need to conceal opaque structures within the body cavity may then explain why glass frogs retain green pigmentation in their dorsal skin (Barnett et al. 2025; Yeager et al. 2026). However, in addition to this translucent green pigmentation, opaque patterns, ranging from small brightly colored spots to large background matching blotches and full‐body reticulations, are found in a wide variety of different glass frog species (Guayasamin et al. 2009). These opaque patches on otherwise translucent frogs could function as background matching, disruptive camouflage, or simply serve to obstruct the view of any internal structures (Barnett et al. 2025). However, little is known about how the presence of these patterns may affect the efficacy of translucent camouflage in glass frogs (Yeager et al. 2026).

In this study, we expand upon Yeager et al. (2026) to test whether opaque dorsal patterns on otherwise translucent prey can improve glass frog camouflage. Specifically, we test whether large spots of opaque green pigmentation can conceal salient internal structures while retaining the camouflaging effect of translucency. To test this hypothesis, we conducted a field predation study where wild predators were presented with model glass frogs that contained internal opaque structures, but which differed in opacity (i.e., opaque and translucent) and dorsal patterning (i.e., with and without spots). We predicted that predation rates would be highest for opaque frogs and when the internal patterns were not entirely concealed, and that the addition of opaque dorsal spots would reduce predation risk.

## 2 Methods

### 2.1 Model Construction

We designed our frog models following methods outlined in Barnett et al. (2020) and Yeager et al. (2026). Each model was made from a mixture of water, unflavored gelatin (Supermaxi, Ecuador), food coloring (Supermaxi, Ecuador), cornstarch (Supermaxi, Ecuador), and modeling clay (Doricolor, Superpaco, Ecuador).

We developed four *model types* which varied in both opacity (O = opaque; T = translucent) and the presence of a spotted pattern on the dorsum (S = spotted; N = unspotted): OS = opaque‐spotted, ON = opaque‐unspotted, TS = translucent‐spotted, TN = translucent‐unspotted (Figure 1). All models included an opaque white dorsolateral stripe which was embedded within the gelatin to represent the opaque internal organs of glass frogs (Yeager et al. 2026). As such, ON and TN were identical to the opaque‐center and translucent‐center models used in Yeager et al. (2026). These spots did not fully hide the clay from view but did partially cover the internal stripe. Pattern treatments were not designed to mimic any specific glass frog species, but rather to examine fundamental questions regarding the role of opaque patterning in translucent camouflage.

**FIGURE 1:**
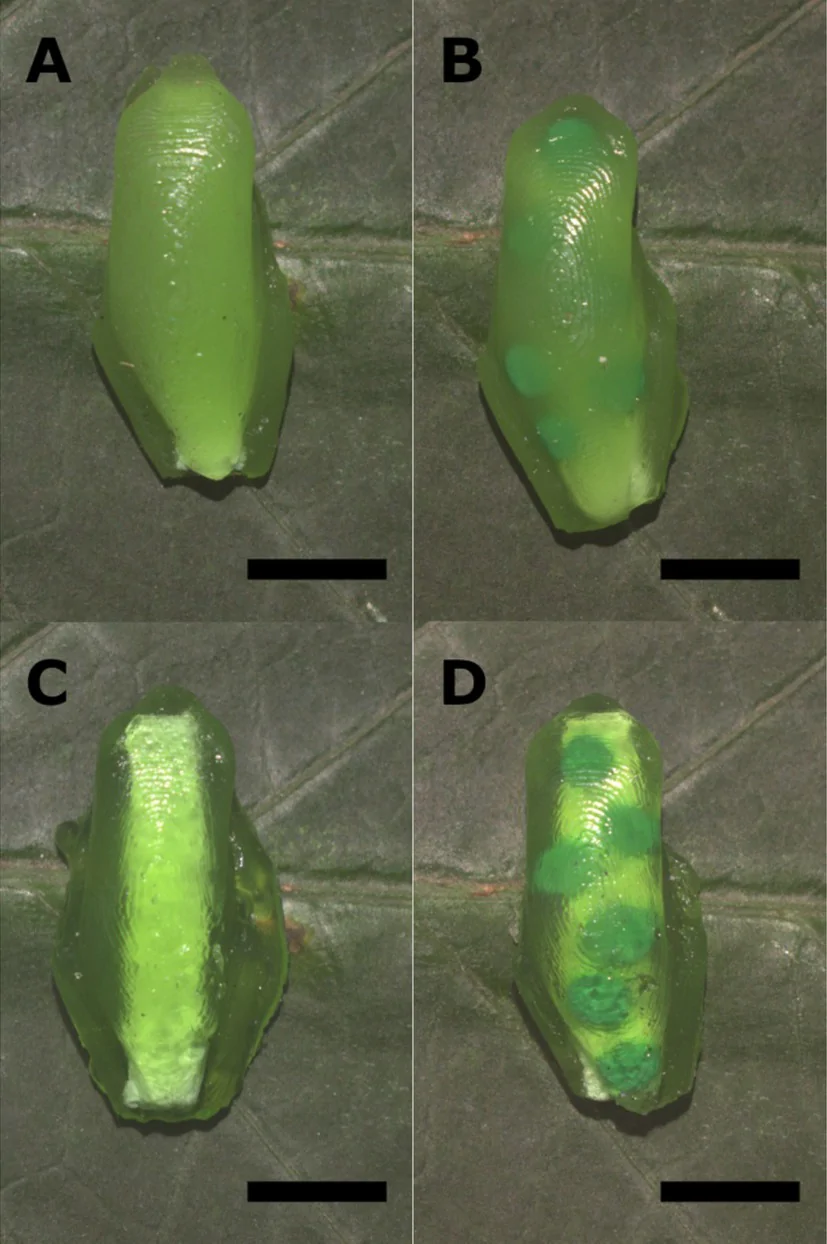
Examples of the experimental treatments. (A) ON = opaque‐unspotted, (B) OS = opaque‐spotted, (C) TN = translucent‐unspotted (c), and (D) TS = translucent‐spotted.

For the model's main gelatin structure, we first dissolved 52.5 g of unflavored gelatin powder into 380 mL of warm water (180 mL cold + 200 mL boiling) which had been dyed green with 0.15 mL (3 drops) of food coloring. Then, to alter the opacity of the gelatin, we added cornstarch powder to the mixture: 0.62 g (1/4 teaspoon) for the translucent models and 5 g (2 teaspoons) for the opaque models.

The opaque internal stripe and the dorsal spotted pattern were both made from modeling clay. To make the internal stripe, we first gave the modeling clay a green tint by mixing 32 g of white modeling clay with 0.7 mL (14 drops) of green food coloring. The clay was then passed through a clay extruder to form strips (56 mm × 4 mm × 1 mm) that were folded in half to create a thick stripe (28 mm long × 8 mm wide × 1 mm deep). To make the dorsal spotted pattern, we mixed 10 g of white modeling clay with 1.5 mL (30 drops) of blue food coloring and 2.0 mL (40 drops) of green food coloring to create a saturated green hue. The modeling clay was divided into 2 mm spheres that were flattened to form circular disks which approximated the size of the spots found in some *Hyalinobatrachium* spp. which are present at the site.

To make the models, the gelatin mixture was poured into custom‐made silicone molds (Smooth‐Sil 940, Smooth‐On Inc., Macungie, PA, USA) based on a 3D‐printed model of a tree frog ( *Phyllomedusa tomopterna* ) in a resting posture (DigitalLife3D, University of Massachusetts; Barnett et al. 2020). The mold was scaled to 25 mm in length to match the body length of the glass frog *Espadarana prosoblepon* (Barnett et al. 2020). For the models with spotted patterns, 6 green disks were added to the bottom of the mold prior to adding the gelatin mixture so that they would be stuck to the dorsal surface of the model. The placement of the spots varied slightly as the gelatin solidified, meaning that each pattern was unique. This variation was independent of treatment and so would not have biased our results. The internal opaque stripe was added to all models by gently pushing the clay strip into the center of the mold immediately after the gelatin mixture had been poured. Finally, the models were transferred to a refrigerator where they could fully solidify. Models were refrigerated for at least 1 h before being removed from the mold and were then stored in resealable plastic bags for no more than 2 days before being used in the experiment. Prior to deployment, each model was removed from the bag so that it would partially desiccate overnight. This meant that the models could absorb ambient humidity from the forest and would adhere to the leaves more strongly.

### 2.2 Experimental Protocol

We conducted our experiment at a site of mature rainforest within the Mashpi‐Tayra Wildlife Refuge (Pichincha, Ecuador) where several species of glass frog are found in sympatry (Yeager et al. 2026). Little is known about the specific predators of glass frogs, but reports suggest that birds (Quiroga‐Carmona and Naveda‐Rodríguez 2014; de Souze et al. 2016), bats (Tuttle and Ryan 1981), snakes (Ortega‐Andrade et al. 2013), and various invertebrates (Hayes 1983) will consume these frogs. As birds are primarily visual predators (Martin 2022) and are therefore likely to have a significant influence on color evolution, we focus on bird predation here. We used the same transect (~780 m in length) along the banks of the Laguna River as used in Yeager et al. (2026) but ~17 months after any similar experiments had been conducted in the area.

We used a randomized block design where the transect was divided into 60 *blocks* that were spaced ~5 m apart along the path. We included the four *model types* (OS, ON, TN, & TS) and, as Yeager et al. (2026) found a significant effect of model height, we placed each *model type* at two different heights: high at ~1.5 m and low at ~0.5 m above the forest floor. Each *block* included one of each of the eight *model type* × *height* combinations (4 *model types* at 2 *heights*). Each model was attached to a living leaf by partially melting the underside of the model with a lighter before pressing it firmly onto the upper surface of the leaf. Within each *block*, models were placed in a random order, out of direct line of sight, at least 1 m apart, and at least 0.5 m from the riverbank.

After deployment we checked the models for signs of attempted predation at 24, 48, and 72 h. Predator identity was determined by the marks left in the gelatin and we recorded all missing, damaged, and surviving models as censored values. The beaks of birds left V‐shaped slices in the gelatin, the incisor teeth of rodents left recognizable parallel lines, the teeth of lizards left a U‐shaped pattern of circular holes with pin teeth marks, and invertebrates (such as ants) left small rough pits or scratches across the surface of the model. We repeated this protocol in two sequential *rounds* along the same transect but with the location and order of models independently randomized between *rounds* (R1: 12–16 December 2025; R2: 17–20 December 2025). In total we deployed 960 models (120 per treatment‐height combination).

### 2.3 Statistical Analysis

We analyzed the rate at which the frog models were predated using a mixed‐effect Cox survival model from the *coxme* v.2.2–22 package (Therneau 2024) in R v.4.5.3 (R Core Team 2026). We analyzed the data twice. Firstly, using all likely predation events from different vertebrate predators (i.e., birds, mammals, and reptiles) as well as all missing models (i.e., those likely removed and consumed by predators). Then, secondly, we used a more conservative estimate of predation, which only included confirmed bird attacks (i.e., those with clear beak marks left in the gelatin). In both cases, frog models which fell to the ground during the experiment, only showed minor scratch marks (likely from invertebrates or falling debris), or remained undamaged after 72 h were included in the analysis as censored values.

Each statistical model included the fixed effects of *model type* (OS, ON, TS, & TN) and *height* (low & high) as well as the random effects of *block* and *round*. To examine whether the effect of *model type* differed according to its *height* we created both an interactive model (*model type* * *height*) and an additive model (*model type* + *height*), and then compared model fit using a Likelihood Ratio test (function *anova*) and the Akaike Information Criterion (AIC, function *AIC*) from base R v.4.5.3. We used the function *cox.zph* from R package *survival* v.3.8–6 (Therneau 2024) to check whether each fixed effect conformed to the proportional hazards assumption and we then used the *Anova* function from R package *car* v.3.1.3 (Fox and Weisberg 2019) to examine the significance of the fixed effects (i.e., *model type* & *height*).

If the main effect of *model type* was significant, we then used the R package *multcomp* v.1.4.30 (Hothorn et al. 2008) to perform pairwise contrasts of a priori interest. To examine whether greater translucency reduced predation risk, we compared the survival of *opaque* and *translucent* models while keeping the spotted pattern the same (i.e., spotted opaque vs. spotted translucent: OS vs. TS & unspotted opaque vs. unspotted translucent: ON vs. TN). To examine whether spotted patterns reduced the risk of predation, we compared attack rates on *spotted* and *unspotted* models while controlling for model translucency (i.e., spotted opaque vs. unspotted opaque: OS vs. ON & spotted translucent vs. unspotted translucent: TS vs. TN).

## 3 Results

### 3.1 All Likely Predation Events

We first analyzed the survival data by including all likely attacks from vertebrate predators regardless of the species (i.e., all marks recognizable as bird, lizard, and mammal attacks) and all missing models (i.e., assuming that missing models were consumed by a vertebrate predator). We found that 493 of the 960 (51%) models met these criteria and showed some evidence of ‘predation’: including 388 (40%) attacked and 105 (11%) missing. When considering the effects of *model type* and *height* on survival, we found that including an interaction term did not significantly improve model fit (*χ* ^2^ = 0.29, df = 3, *p* = 0.962) and that an additive model was a better fit to the data than an interactive model (AIC: additive = 6213, interactive = 6219). We therefore interpreted the effects of *model type* and *height* independently. Model survival did not significantly differ according to *height* (*χ* ^2^ = 1.84, df = 1, *p* = 0.175) but there was a significant effect of *model type* (*χ* ^2^ = 55.40, df = 3, *p* < 0.001; Figure 2A). We then performed pairwise contrasts of a priori interest to investigate the role of model translucency while controlling for pattern (OS vs. TS & ON vs. TN) and spot pattern while controlling for translucency (OS vs. ON & TS vs. TN). We found no significant effect of model translucency (OS = TS: *z* = −0.68, *p* = 0.879 | ON = TN: *z* = 0.68, *p* = 0.881). Whereas there was a significant effect of spot pattern with spotted models having lower predation risk than unspotted models regardless of whether the underlying model was opaque (OS<ON: *z* = −6.03, *p* < 0.001) or translucent (TS<TN: *z* = −4.52, *p* < 0.001).

**FIGURE 2:**
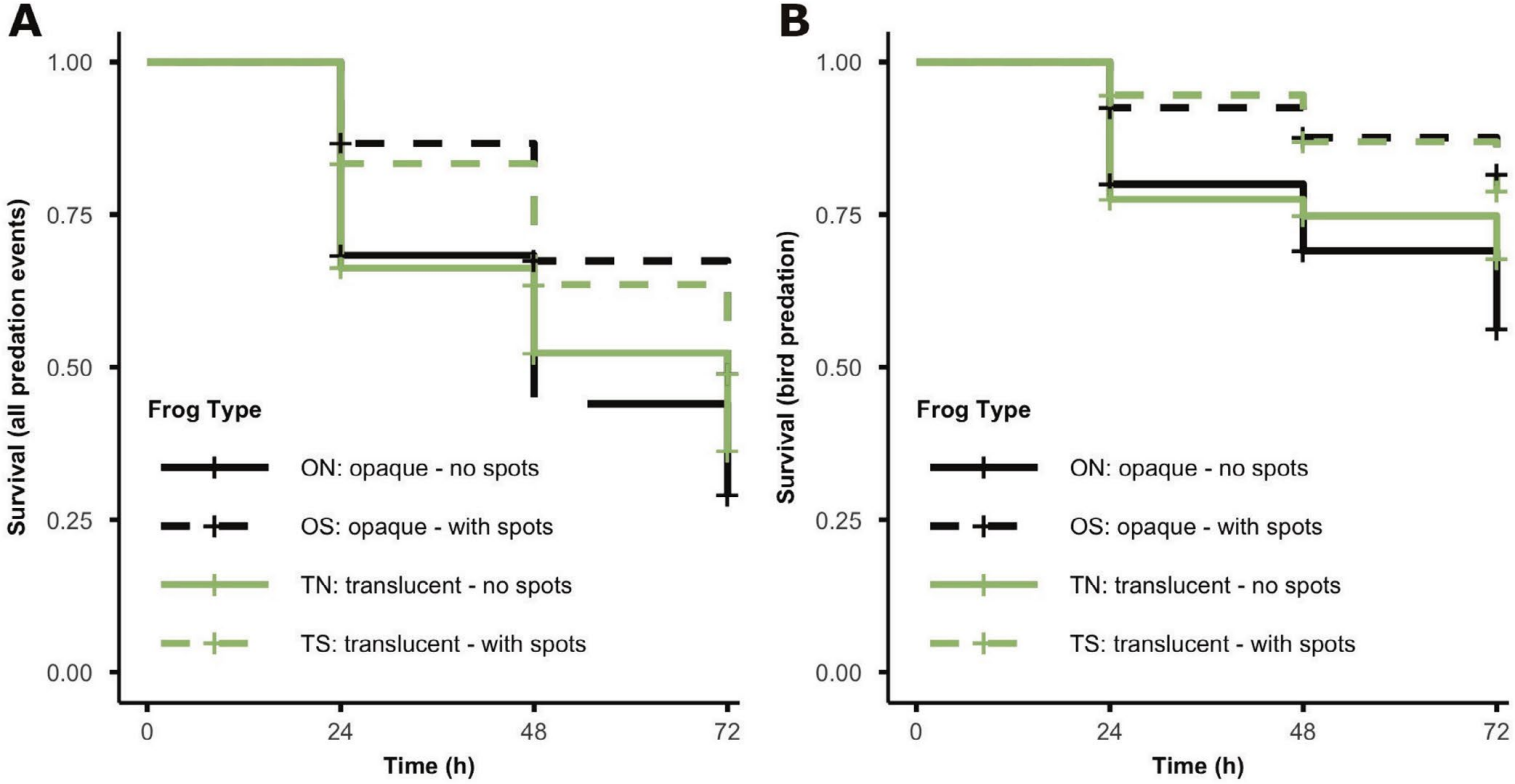
Survival analysis from the field predation study. Survival curves for the effect of *model type* (color: Black = opaque, green = translucent; line type: Solid = no spots, dashed = spotted | ON = opaque—no spots, OS = opaque—spots, TN = translucent—no spots, TS = translucent—spots). (A) Including all likely predation events and missing models. (B) Only including confirmed bird attacks. In both analyses, there was no significant effect of model translucency (OS = TS & ON = TN) but for both opaque and translucent models, those with spots had a lower risk of predation than those without spots (OS<ON & TS<TN).

### 3.2 Confirmed Bird Predation Only

We next focused on the confirmed bird attacks as a conservative estimate of attack rates from visually orientated predators. We found that 219/960 models (23%) showed identifiable evidence of bird attacks (i.e., beak marks). When analyzing predation risk, we found that, in both the additive and interactive models, *model type* did not conform to the proportional hazards assumption (additive: *χ* ^2^ = 9.68, df = 3, *p* = 0.021 | interactive: *χ* ^2^ = 9.95, df = 3, *p* = 0.019). Although this is likely due to a small number of events in some time periods, it is important to note that deviation from proportional hazards may indicate that the risk of predation changes as the time from deployment increases. Including a time dependent covariate did not improve model fit and so here we report the simpler model where risk is averaged across the 72‐h time period. As our hypotheses concern overall predation risk, rather than differences in when predation occurs, this does not significantly affect the interpretation of our data.

We found that the interaction between *model type* and *height* was not significant (*χ* ^2^ = 0.36, df = 3, *p* = 0.948; model AIC: additive = 2765, interactive = 2770) and so we interpreted the effects of *model type* and *height* separately. There was no significant effect of *height* on survival (*χ* ^2^ = 2.14, df = 1, *p* = 0.144), whereas predation risk did differ according to *model type* (*χ* ^2^ = 55.78, df = 3, *p* < 0.001; Figure 2B). Pairwise contrasts found no significant effect of translucency (OS = TS: *z* = −0.57, *p* = 0.925 | ON = TN: *z* = 0.67, *p* = 0.884) but that models with spotted patterns had lower risk of predation than unspotted models (OS<ON: *z* = −5.93, *p* < 0.001 | TS<TN: *z* = −4.62, *p* < 0.001).

## 4 Discussion

The glass frogs are a classic example of transparent camouflage, but rather than being completely see‐through these frogs are a translucent green and frequently have opaque spotted patterns on their dorsal surface (Barnett et al. 2020, 2025; Guayasamin et al. 2020; Taboada et al. 2022). Previous work, using gelatin models, suggests that when compared to opaque frogs, translucent frogs more accurately matched the luminance of the underlying substrate and experienced significantly lower predation risk (Barnett et al. 2020). However, a follow‐up study found that the benefits of translucent camouflage were significantly weakened when opaque and saliently colored structures (representing the opaque viscera of glass frogs) were visible within the gelatin models (Yeager et al. 2026).

Here, we built on Yeager et al. (2026), to explicitly test whether opaque (cryptically colored) spots on the dorsum can reduce the detectability of opaque internal structures and recover the benefits of translucent camouflage described by Barnett et al. (2020). We found that gelatin models with opaque green spots have lower predation risk than unspotted models where the internal structures remained entirely visible. However, as with Yeager et al. (2026), we found no evidence that the translucency of the surrounding gelatin affected predation risk.

Taken together with this previous work, our data suggest that the presence of opaque structures within the body (e.g., the viscera) can reduce the survival benefits of translucent camouflage (Barnett et al. 2020; Yeager et al. 2026) but that opaque patches of dorsal pigmentation (i.e., dorsal spots) can significantly reduce the heightened predation risk associated with visible internal structures (Yeager et al. 2026). However, dorsal spots did not restore the cryptic effects of translucency (Barnett et al. 2020). This discrepancy may have resulted from differences in the degree of translucency between experiments, but as all models were made using the same recipe, these data may instead suggest that the opaque internal structures, even when partially covered by spots, may have been too salient to be effectively counterbalanced by the translucent areas (Yeager et al. 2026).

One mechanism by which partially transparent patterns may be beneficial is disruptive camouflage through differential blending. Here different components of the pattern match different parts of the background such that the target appears to be broken up into a series of unrecognizable features (Stevens and Merilaita 2008; Merilaita et al. 2017). This strategy has previously been suggested to explain the wings of moths that include both transparent and opaque patches (Barnett et al. 2025). For example, Costello et al. (2020) found that small transparent patches (representing holes) on the wings of otherwise cryptically colored moths could reduce detectability through surface disruption, and Arias et al. (2021) found that partial transparency along the edge of the wing could provide edge disruption.

Our findings contrast with these studies, with our data instead suggesting that there was no benefit associated with being translucent when opaque patches (both salient internal stripes and cryptic dorsal spots) were added to the gelatin frog models. This could be because our translucent patches were comparatively too small, because the opaque regions did not match the background color well enough, or because the pattern edges may have exceeded the range of contrast boundaries found in the substrate. Consequently, our finding that cryptic dorsal spots can improve concealment may be best explained by the dorsal pigmentation partially blocking the more salient internal structures from view and reducing the apparent size of this conspicuous stripe. This effect would be more akin to increasing the area of effective background matching than to the disruptive effects described in the moth experiments (Costello et al. 2020; Arias et al. 2021).

Indeed, the background may be too homogeneous to allow for effective disruptive camouflage even if the internal stripe had matched the background. The simple, largely patternless structure of living leaves may not provide sufficient heterogeneity in color and luminance for differential blending (Price et al. 2019). In previous work looking at transparent disruptive camouflage, the background was more complex with variation in both color, texture, and depth (i.e., bramble bushes—Costello et al. 2020; oak tree bark—Arias et al. 2021). Given the homogeneous nature of the glass frogs' natural background, alternative mechanisms of camouflage may be more effective. For example, edge diffusion in which a smooth gradient from opaque to translucent, rather than a sharp boundary between translucent and opaque patches, blends the target into the background (Webster et al. 2015; Barnett et al. 2020, 2025). Small opaque spots overlayed onto these graduated pattern may then mimic imperfections on the leaf, such as damage or fungal growth (Barnett et al. 2025). Background complexity is an important factor affecting the efficacy of opaque camouflage (Merilaita et al. 2017; Cuthill 2019), but more work is needed to understand how background structure affects the evolution and detectability of partial and imperfectly transparent organism.

We also found no evidence that model height affected the survival of our model frogs. In Yeager et al. (2026), models which were placed on lower perches (~0.5 m above the forest floor) were more likely to be attacked than models placed higher up (~1.5 m above the forest floor). This effect of height was speculatively attributed to predation from terrestrial birds (e.g., guans) foraging on the forest floor. The difference between these two studies may reflect seasonal differences in predation pressure, with this study being conducted in the wetter season (December) while previous work was undertaken during the drier season (June; Yeager et al. 2026). However, we lack precise data on which specific predator species are attacking these models and how predator behavior may change depending on model height, time of day, and season, or if the predator community remains constant over time.

Animal transparency has long been recognized as a form of concealment (Johnsen 2001; Bagge 2019; Cuthill 2019; Barnett et al. 2025). However, although the benefits of being highly see‐through may be intuitively understood, transparency is a variable trait with diversity in both the degree of light transmission and in how transparent regions may be combined with opaque pattern components (Johnsen 2001; Bagge 2019; Barnett et al. 2025). The presence of opaque patches on otherwise transparent species may initially appear to be maladaptive, but recent evidence suggests that combining transparent and opaque regions can facilitate a range of different defensive strategies, from camouflage to aposematism (Barnett et al. 2025).

Opaque dorsal color patterns are common in many different species of glass frog, ranging from small and brightly colored spots to large seemingly cryptic blotches and reticulations (Guayasamin et al. 2020), but little is known about how they may affect camouflage and whether they increase or decrease the survival benefit conferred by translucency. Our data suggest that the opaque color patterns of glass frogs may function to conceal opaque and highly salient internal organs from view. However, we found no evidence that combining these background matching patterns with translucent patches had any additional advantage over fully opaque patterns.

Glass frogs can offer important insights into the evolution and function of transparency; however, much remains understudied. For example, in light of our findings here, more work is warranted to investigate the full range of interspecific diversity found among glass frog species, including variation in transparency, body size, dorsal color patterns, and microhabitat use. Such data can help to more accurately represent the diversity and complexity of anti‐predatory strategies that may be facilitated by transparent or translucent body parts and highlight where transparent camouflage may have an advantage over opaque coloration.

These data reveal that transparency does not always confer effective concealment and that camouflage may be limited by both the degree of transparency and the efficacy of opaque background matching. Future work is needed to understand how partial or imperfect transparency may provide camouflage, how transparent camouflage may be affected by characteristics (such as homogeneity) of the background, and how variation in the size, shape, placement, and opacity of transparent/translucent elements affects detectability and predation risk under natural conditions and across transparent taxa.

## Author Contributions

**Justin Yeager:** conceptualization (equal), funding acquisition (lead), investigation (equal), methodology (equal), project administration (equal), resources (lead), supervision (equal), validation (equal), visualization (equal), writing – original draft (equal), writing – review and editing (equal). **Leonardo Ávila:** data curation (lead), investigation (lead), writing – review and editing (equal). **James B. Barnett:** conceptualization (equal), formal analysis (lead), methodology (equal), validation (equal), visualization (equal), writing – original draft (equal), writing – review and editing (equal).

## Funding

Funding was provided to J.Y. by the Universidad de las Américas, Quito, Ecuador (UDLA: 483.A.XIV.24).

## Ethics Statement

Work was conducted with the permission of the Ministerio del Ambiente, Ecuador (permits: MAATE‐ARSFC‐2025‐0345).

## Conflicts of Interest

The authors declare no conflicts of interest.

## Data Availability

Raw data and analysis code is available from Dryad: https://doi.org/10.5061/dryad.sqv9s4nkr.

## References

[ece374623-bib-0001] Angiolani‐Larrea, F. N. , A. Valencia‐Aguilar , M. Garrido‐Priego , et al. 2025. “Is There a Right Place? The Effect of Within‐Leaf Clutch Location on Offspring Survival in a Glassfrog.” PLoS One 20, no. 4: e0309642. 10.1371/journal.pone.0309642. PMC11990747 40215211

[ece374623-bib-0002] Arias, M. , L. Leroy , C. Madec , et al. 2021. “Partial Wing Transparency Works Better When Disrupting Wing Edges: Evidence From a Field Experiment.” Journal of Evolutionary Biology 34: 1840–1846. 10.1111/jeb.13943. 34601773

[ece374623-bib-0003] Bagge, L. E. 2019. “Not as Clear as It May Appear: Challenges Associated With Transparent Camouflage in the Ocean.” Integrative and Comparative Biology 59: 1653–1663. 10.1093/icb/icz066. 31141119

[ece374623-bib-0004] Barnett, J. B. , C. Michalis , H. M. Anderson , et al. 2020. “Imperfect Transparency and Camouflage in Glass Frogs.” Proceedings of the National Academy of Sciences of the United States of America 117: 12885–12890. 10.1073/pnas.1919417117. PMC7293656 32457164

[ece374623-bib-0005] Barnett, J. B. , J. Yeager , and K. Kjernsmo . 2025. “Animal Transparency: How Should We Define Form and Function?” Functional Ecology 39: 929–941. 10.1111/1365-2435.70009.

[ece374623-bib-0006] Cisneros‐Heredia, D. F. , and R. W. Mcdiarmid . 2007. “Revision of the Characters of Centrolenidae (Amphibia: Anura: Athesphatanura), With Comments on Its Taxonomy and the Description of New Taxa of Glassfrogs.” Zootaxa 1572: 1–82. 10.11646/zootaxa.1572.1.1.

[ece374623-bib-0007] Costello, L. M. , N. E. Scott‐Samuel , K. Kjernsmo , and I. C. Cuthill . 2020. “False Holes as Camouflage.” Proceedings of the Royal Society B 287: 20200126. 10.1098/rspb.2020.0126. PMC7126085 32156219

[ece374623-bib-0008] Cuthill, I. C. 2019. “Camouflage.” Journal of Zoology 308: 75–92. 10.1111/jzo.12682.

[ece374623-bib-0009] de Souze, R. D. R. , M. Moroti , J. Briet , and I. F. Machado . 2016. “ *Vitreorana uranoscopa* . Predation by *Attila rufus* .” Herpetological Review 47: 651.

[ece374623-bib-0010] Fox, J. , and S. Weisberg . 2019. An R Companion to Applied Regression. Third ed. Sage. https://www.john‐fox.ca/Companion/.

[ece374623-bib-0011] Guayasamin, J. M. , S. Castroviejo‐Fisher , L. Trueb , J. Ayarzagüena , M. Rada , and C. Vilà . 2009. “Phylogenetic Systematics of Glassfrogs (Amphibia: Centrolenidae) and Their Sister Taxon *Allophryne ruthveni* .” Zootaxa 2100: 1–97. 10.11646/zootaxa.2100.1.1.

[ece374623-bib-0012] Guayasamin, J. M. , D. F. Cisneros‐Heredia , R. W. McDiarmid , P. Peña , and C. R. Hutter . 2020. “Glassfrogs of Ecuador: Diversity, Evolution, and Conservation.” Diversity 12: 222. 10.3390/d12060222.

[ece374623-bib-0014] Hayes, M. P. 1983. “Predation on the Adults and Pehatching Stages of Glass Frogs (Centrolenidae).” Biotropica 15: 74–76. 10.2307/2388005.

[ece374623-bib-0015] Hothorn, T. , F. Bretz , and P. Westfall . 2008. “Simultaneous Inference in General Parametric Models.” Biometrical Journal 50: 346–363. 10.1002/bimj.200810425. 18481363

[ece374623-bib-0016] Johnsen, S. 2001. “Hidden in Plain Sight: The Ecology and Physiology of Organismal Transparency.” Biological Bulletin 201: 301–318. 10.2307/1543609. 11751243

[ece374623-bib-0017] Martin, G. R. 2022. “Avian Vision.” Current Biology 32: R1079–R1085. 10.1016/j.cub.2022.06.065. 36283369

[ece374623-bib-0018] Merilaita, S. , N. E. Scott‐Samuel , and I. C. Cuthill . 2017. “How Camouflage Works.” Philosophical Transactions of the Royal Society 372: 20160341. 10.1098/rstb.2016.0341. PMC5444062 28533458

[ece374623-bib-0019] Ortega‐Andrade, H. M. , O. Rojas‐Soto , and C. Paucar . 2013. “Novel Data on the Ecology of *Cochranella mache* (Anura: Centrolenidae) and the Importance of Protected Areas for This Critically Endangered Glassfrog in the Neotropics.” PLoS One 8: e81837. 10.1371/journal.pone.0081837. PMC3855356 24339973

[ece374623-bib-0020] Price, N. , S. Green , J. Troscianko , T. Tregenza , and M. Stevens . 2019. “Background Matching and Disruptive Coloration as Habitat‐Specific Strategies for Camouflage.” Scientific Reports 9: 7840. 10.1038/s41598-019-44349-2. PMC6534618 31127182

[ece374623-bib-0021] Quiroga‐Carmona, M. , and A. Naveda‐Rodríguez . 2014. “Crested Quetzal ( *Pharomachrus antisianus* ) Preying on a Glassfrog (Anura, Centrolenidae) in Sierra de Perijá, Northerwestern Venezuela.” Revista Brasileira de Ornitologia 22: 419–421. 10.1007/BF03544279.

[ece374623-bib-0500] R Core Team . 2026. R: A Language and Environment for Statistical Computing. R Foundation for Statistical Computing.

[ece374623-bib-0023] Stevens, M. , and S. Merilaita . 2008. “Defining Disruptive Coloration and Distinguishing Its Functions.” Philosophical Transactions of the Royal Society, B: Biological Sciences 364: 481–488. 10.1098/rstb.2008.0216. PMC2674077 18990673

[ece374623-bib-0024] Taboada, C. , J. Delia , M. Chen , et al. 2022. “Glassfrogs Conceal Blood in Their Liver to Maintain Transparency.” Science 378: 1315–1320. 10.1126/science.abl6620. PMC9984244 36548427

[ece374623-bib-0025] Therneau, T. M. 2024. “coxme: Mixed Effects Cox Models.” R Package v. 2.2‐22. 10.32614/CRAN.package.coxme.

[ece374623-bib-0026] Tuttle, M. D. , and M. J. Ryan . 1981. “Bat Predation and the Evolution of Frog Vocalisations in the Neotropics.” Science 214: 677–678. 10.1126/science.214.4521.677. 17839660

[ece374623-bib-0027] Webster, R. J. , J. G. J. Godin , and T. N. Sherratt . 2015. “The Role of Body Shape and Edge Characteristics on the Concealment Afforded by Potentially Disruptive Marking.” Animal Behaviour 104: 197–202. 10.1016/j.anbehav.2015.03.027.

[ece374623-bib-0028] Yeager, J. , A. Robison , C. D. Wade , and J. B. Barnett . 2026. “High Contrast Markings Can Negate the Benefits of Transparent Camouflage.” Ecology and Evolution 16: e73490. 10.1002/ece3.73490. PMC13068497 41970357

